# *Para-* and *Ortho*-Substitutions Are Key Determinants of Polybrominated Diphenyl Ether Activity toward Ryanodine Receptors and Neurotoxicity

**DOI:** 10.1289/ehp.1002728

**Published:** 2010-11-24

**Authors:** Kyung Ho Kim, Diptiman D. Bose, Atefeh Ghogha, Joyce Riehl, Rui Zhang, Christopher D. Barnhart, Pamela J. Lein, Isaac N. Pessah

**Affiliations:** 1 Department of Molecular Biosciences, School of Veterinary Medicine and; 2 Center for Children’s Environmental Health, University of California–Davis, Davis, California, USA

**Keywords:** calcium, hydroxylated PBDE, methoxylated PBDE, neurotoxicity, polybrominated biphenyl ether (PBDE), ryanodine receptor (RyR)

## Abstract

**Background:**

Polybrominated diphenyl ethers (PBDEs) are widely used flame retardants that bioaccumulate in human tissues. Their neurotoxicity involves dysregulation of calcium ion (Ca^2+^) signaling; however, specific mechanisms have yet to be defined.

**Objective:**

We aimed to define the structure–activity relationship (SAR) for PBDEs and their metabolites toward ryanodine receptors type 1 (RyR1) and type 2 (RyR2) and to determine whether it predicts neurotoxicity.

**Methods:**

We analyzed [^3^H]ryanodine binding, microsomal Ca^2+^ fluxes, cellular measurements of Ca^2+^ homeostasis, and neurotoxicity to define mechanisms and specificity of PBDE-mediated Ca^2+^ dysregulation.

**Results:**

PBDEs possessing two *ortho*-bromine substituents and lacking at least one *para*-bromine substituent (e.g., BDE-49) activate RyR1 and RyR2 with greater efficacy than corresponding congeners with two *para-*bromine substitutions (e.g., BDE-47). Addition of a methoxy group in the free *para* position reduces the activity of parent PBDEs. The hydroxylated BDEs 6-OH-BDE-47 and 4′-OH-BDE-49 are biphasic RyR modulators. Pretreatment of HEK293 cells (derived from human embryonic kidney cells) expressing either RyR1 or RyR2 with BDE-49 (250 nM) sensitized Ca^2+^ flux triggered by RyR agonists, whereas BDE-47 (250 nM) had negligible activity. The divergent activity of BDE-49, BDE-47, and 6-OH-BDE-47 toward RyRs predicted neurotoxicity in cultures of cortical neurons.

**Conclusions:**

We found that PBDEs are potent modulators of RyR1 and RyR2. A stringent SAR at the *ortho* and *para* position determined whether a congener enhanced, inhibited, or exerted nonmonotonic actions toward RyRs. These results identify a convergent molecular target of PBDEs previously identified for noncoplanar polychlorinated biphenyls (PCBs) that predicts their cellular neurotoxicity and therefore could be a useful tool in risk assessment of PBDEs and related compounds.

Worldwide use of chemically stable polybrominated diphenyl ethers (PBDEs) as flame retardants in consumer products has increased since the 1970s (Costa et al. 2008). Levels of PBDEs in the environment (Hale et al. 2003; Law et al. 2006; Oros et al. 2005) and tissues of invertebrates, fish, birds, and mammals have increased in the last 30 years (Yogui and Sericano 2009). In humans, PBDEs have been detected in numerous tissues, with serum concentrations reported as high as 50 ng/g lipid (Petreas et al. 2003). PBDE concentrations in breast milk doubled every 5 years between 1972 and 1997 (Rahman et al. 2001; She et al. 2007). The predominant routes of exposure are diet and inhalation (Allen et al. 2007; Costa et al. 2008; Rose et al. 2010; Schecter et al. 2005). PBDEs with less than five bromine substitutions, such as 2,2′,4,4′-tetrabromodiphenyl ether (BDE-47) and 2,2′,4,4′,5-pentabromodiphenyl ether (BDE-99), have attracted the greatest concern because they show the greatest bioaccumulation (Darnerud et al. 2001) and can be formed by debromination of higher-substituted PBDEs (Gerecke et al. 2005; Stapleton et al. 2006; Stapleton and Dodder 2008).

PBDEs share structural similarities to a subset of the polychlorinated biphenyls (PCBs) that have more than one chloro-substituent in the *ortho* position, in particular, noncoplanarity of their phenyl rings. Like the *ortho*-substituted PCBs (Denison and Nagy 2003; Van den Berg et al. 2006), PBDEs (Peters et al. 2004) have very weak or negligible activity at the aryl hydrocarbon receptor, but disrupt calcium ion (Ca^2+^) signaling in several cell types (Dingemans et al. 2008, 2010). One mechanism by which *ortho-*substituted PCBs disrupt Ca^2+^ signaling and contribute to neurotoxicity is via interactions with ryanodine receptors (RyRs) (Pessah et al. 2010; Wong and Pessah 1996). For example, 2,2′,3,5′,6-pentachlorobiphenyl (PCB-95) at nanomolar concentrations sensitizes RyR Ca^2+^ channels in rat hippocampal microsomal preparations (Pessah et al. 2010; Wong et al. 1997a), and alters neuroplasticity in rat hippocampal slices (Kim et al. 2009). Developmental exposure to PCB-95 or Aroclor 1254 results in behavioral abnormalities in rats and altered patterns of RyR expression in brain (Schantz et al. 1997; Wong et al. 1997b; Yang et al. 2009). In rats exposed perinatally, PCB-95 alters the balance of excitatory/inhibitory currents and disrupts the tonotopic map of the primary auditory cortex (Kenet et al. 2007). Identification of RyRs as a relevant molecular target in PCB neurotoxicity may be generalized to related noncoplanar persistent organic pollutants such as PBDEs. Evidence of a convergent mechanism by which PCBs and PBDEs dysregulate RyRs would be of toxicological significance considering that these tightly regulated Ca^2+^ channels reside within specialized regions of sarcoplasmic/endoplasmic reticulum (SR/ER) membranes where they contribute to and regulate essential aspects of Ca^2+^ signaling (Pessah et al. 2010).

In the present study we examined the structure–activity relationship (SAR) of PBDEs and their metabolites toward the two major RyR isoforms found in the brain and striated muscle (RyR1 and RyR2). Our findings indicate that PBDEs are potent modulators of both RyR isoforms. *Para*- and *ortho*-substitutions are a key structural determinant of this activity and are predictive of their neurotoxicity.

## Materials and Methods

### Materials

We purchased neat certified 2,2′-dibromodiphenyl ether (BDE-4), 4,4′-dibromodiphenyl ether (BDE-15), 2,2′,4-tribromodiphenyl ether (BDE-17; 98.4% pure), 2,2′,3,4′-tetrabromodiphenyl ether (BDE-42; 100% pure), BDE-47 (100% pure), 2,2′,4,5′-tetrabromodiphenyl ether (BDE-49; 100% pure), the hydroxylated BDEs 6-OH-BDE-47 (98.6% pure) and 4′-OH-BDE-49 (97.8% pure), and PCB-95 (99.1 ± 4% pure) from AccuStandard (New Haven, CT) and verified purity and composition by gas chromatography-mass spectrometry through the Superfund Research Program Analytical Core. Methoxy-derivatives were provided by Å. Bergman (Stockholm University, Stockholm, Sweden). [^3^H]Ryanodine ([^3^H]Ry, specific activity 60 Ci/mmol) was obtained from Perkin-Elmer New England Nuclear (Wilmington, DE). 4-Chloro-*m*-cresol (4-CmC), diphenyl ether (DE), rapamycin, and Ca^2+^ ionophore A23187 were purchased from Sigma-Aldrich (St. Louis, MO, USA).

### Animal use

All animal procedures were performed under protocol approved by the University of California–Davis Institutional Animal Care and Use Committee.

### Membrane preparations

Microsomal membrane preparations enriched in RyR1 or RyR2 were isolated from skeletal muscle and heart, respectively, from male New Zealand White rabbits (Charles River Laboratories, Hollister, CA) as described previously (Mack et al. 1992; Zimanyi and Pessah 1991). We prepared brain microsomes from mouse neocortical tissue as described previously for the rat (Wong et al. 1997a).

### [^3^H]Ry binding assays

We determined the amount of specific [^3^H]Ry binding to microsomal membranes in the absence and presence of PBDE or their metabolites, a measure of how RyR activity is affected, as previously described (Pessah et al. 2009). See Supplemental Material (doi:10.1289/ehp.1002728) for details. At least eight concentrations of each congener were tested in triplicate in at least three independent binding assays. The concentration–response curves were analyzed by curve fitting using GraphPad Prism Software (version 5; GraphPad Software, La Jolla, CA).

### Microsomal Ca^2+^ flux measurements

We measured PBDE-induced release of Ca^2+^ actively accumulated by microsomal vesicles in the presence of ATP as previously described (Pessah et al. 2006). See Supplemental Material (doi:10.1289/ehp.1002728) for details. Initial Ca^2+^ release rates were calculated for at least eight PBDE concentrations, including vehicle control. The half-maximal rates of Ca^2+^ release (EC_50_) were determined by curve fitting of three experiments.

### Ca^2+^ imaging of HEK293 cells

We followed the method for growing human embryonic kidney [human embryonic kidney 293 (HEK293); RyR*^null^*] cells in culture and the protocols for transfection and selection protocols for HEK293 cells that stably express RyR1 (HEK293*^RyR1^*) or RyR2 (HEK293*^RyR2^*) as described previously (Pessah et al. 2009). Sixteen hours after introduction of vehicle or PBDE, cells were loaded with the Ca^2+^ indicator Fluo-4 to measure cytoplasmic Ca^2+^ changes in response to challenges with RyR activators caffeine or 4-chloro-*m*-cresol (4-CmC). See Supplemental Material (doi:10.1289/ehp.1002728) for additional details.

### Primary culture of mouse and rat cortical neurons

High-density cultures of cortical neurons were dissociated from postnatal day 0–2 Sprague-Dawley rats (Charles River Laboratories) for electrophysiological measurements (Yang et al. 2009) or C57BL/6J (B6) mice (Charles River Laboratories) for neuronal viability measurements as previously described (Chen et al. 2010). See Supplemental Material (doi:10.1289/ehp.1002728) for details.

### Multielectrode array (MEA) recording and data analysis

MEAs with a central 0.88-mm^2^ recording matrix of 64 multielectrodes (Med-P545A; AutoMate Scientific, Berkeley, CA) were precoated with poly-l-lysine (0.5 mg/mL, Sigma Chemical Co., St. Louis, MO) and laminin (10 μg/mL, Invitrogen, Carlsbad, CA, USA). Dissociated rat cortical cells were plated onto the MEAs at a density of 1 × 10^5^ cells/MEA. Cultures were maintained in Neurobasal-A (Invitrogen) supplemented with B27 (Invitrogen) as previously described (Wayman et al. 2006). Half of the medium was replaced twice weekly with fresh Neurobasal-A containing B27. At 21 days *in vitro*, cortical neurons grown on MEAs were placed into the MED 64CH Integrated Amplifier interface and spike activity was recorded using Mobius software (both from AutoMate Scientific). Baseline activity was recorded for 10 min at 37°C. Cultures were then exposed to vehicle by adding 1 μL DMSO into MEA cultures containing 1 mL culture medium (final 0.1% DMSO), and activity was recorded for 10 min. Subsequently, cultures were exposed to either BDE-47 or BDE-49 by adding 1 μL 1,000× stock solution in DMSO to the well. Cultures were sequentially exposed to increasing concentrations of PBDE, and activity was recorded for 10 min after each addition. We analyzed spontaneous spike activity using the Spike Sorting and DC filter applications in Mobius. Spikes greater than three times the baseline noise were scored. The number of spikes per 10-min recording session was determined (for electrodes showing activity), and the mean spike number per MEA was calculated. Three MEAs from three independent dissections were analyzed per PBDE.

### Tetrazolium-based MTS assay

We performed the MTS [3-(4,5-dimethylthiazol-2-yl)-5-(3-carboxymethoxyphenyl)-2-(4-sulfophenyl)-2H-tetrazolium] assay to test cell viability in cultured mouse cortical neurons after 48-hr exposures to BDE-47, BDE-49 or 6-OH-BDE-47 using the CellTiter 96 Cell Proliferation Assay kit (Promega, Madison, WI) as previously described (Chen et al. 2010). See Supplemental Material (doi:10.1289/ehp.1002728) for additional details. Experiments were performed on three independent cultures, with each vehicle or PBDE concentration replicated in four wells. Differences from vehicle were tested using two-way analysis of variance (ANOVA) with post hoc analysis.

## Results

### *Ortho*-bromine substitution is required for PBDE activity toward RyR1

**Figure 1:**
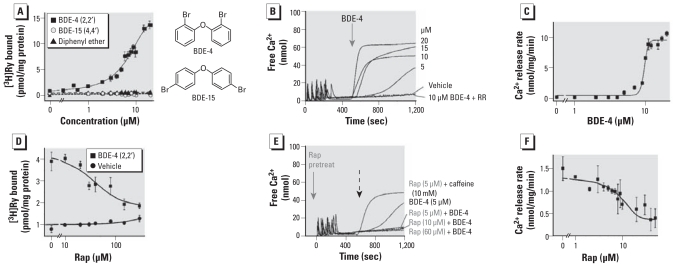
*Ortho*-bromines are essential for PBDE activity toward FKBP12-RyR1 complex. Abbreviations: Rap, rapamycin; RR, ruthenium red. (*A*) Microsomes enriched in RyR1 protein were incubated in the presence of either BDE-4 or BDE-15 in [^3^H]Ry binding buffer. (*B*) Addition of 20 μM BDE-4 (arrow) to microsomal vesicles subsequent to SERCA-dependent Ca^2+^ accumulation in the presence of ATP (loading phase) resulted in net Ca^2+^ release. Inhibition of RyR1 by 1μM RR blocked the Ca^2+^ release triggered by BDE-4 (10 μM). (*C*) Concentration–effect curve for BDE-4–triggered Ca^2+^ release (EC_50_ = 9.6 ± 0.2 μM; *n* = 4). Addition of vehicle to the Ca^2+^-loaded vesicles did not cause Ca^2+^ release (Vehicle). (*D*) Concentration-dependent inhibition of BDE-4–enhanced [^3^H]Ry binding to RyR1 by Rap (5 μM BDE-4 with 0–200 μM Rap); Rap alone had negligible effects on [^3^H]Ry binding to RyR1. *n* = 3 independent experiments in triplicate. (*E*) Pretreatment of microsomal vesicles with Rap 2 min before loading vesicle with Ca^2+^ (arrow) inhibited Ca^2+^ release triggered by subsequent addition (dotted arrow) of 5 μM BDE-4 but did not inhibit caffeine-induced Ca^2+^ release. (*F*) Concentration–effect relationship of Rap inhibition of BDE-4 triggered Ca^2+^ release; *n* = 4.

We first tested the minimal structural requirement for PBDE activity toward RyR1 using [^3^H]Ry binding analysis to microsomes. Neither unsubstituted diphenyl ether nor BDE-15 (< 20 μM) altered the amount of specific binding of [^3^H]Ry to RyR1, a measure of receptor activation (Pessah et al. 1987). In contrast, BDE-4 significantly enhanced specific binding of [^3^H]Ry to RyR1, increasing occupancy 14-fold at 20 μM when measured with suboptimal Ca^2+^ in the assay buffer (Figure 1A). This finding indicates that BDE-4 is an efficacious activator of RyR1 when measured under buffer conditions that promote a closed-channel conformation.

Antipyrylazo-III is a low-affinity membrane impermeable dye that allows quantification of Ca^2+^ fluxes across vesicles (Baylor et al. 1983). Spectroscopic detection of antipyrylazo-III absorbance measures changes in free Ca^2+^ in the extravesicular solution. Ca^2+^ was actively loaded into microsomes via the SR/ER Ca^2+^ ATPase (SERCA) by bolus additions of Ca^2+^ to the assay buffer containing ATP (Ca^2+^-loading phase). After the loading phase was complete (the dye signal re-established baseline), addition of BDE-4 (1–20 μM) elicited a net release of Ca^2+^ (net Ca^2+^ efflux; Figure 1B). The PBDE EC_50_ value was 12.0 ± 0.8 μM under conditions that mimic the resting state of most mammalian cells (i.e., ~ 100 nM free Ca^2+^ at the cytoplasmic face of RyR1). BDE-4–triggered Ca^2+^ release was fully blocked by ruthenium red (RR; 1 μM), an RyR blocker (Figure 1B). EC_50_ values were calculated from plots of the initial rate of Ca^2+^ release as a function of BDE-4 concentration (Figure 1C). BDE-15 and unsubstituted diphenyl ether at concentrations < 20 μM did not elicit detectable release of accumulated Ca^2+^ in the microsomal transport assay (not shown).

Activation of the Ca^2+^ channel by *ortho*-substituted PCBs requires an intact association of RyR1 with its accessory protein FK506-binding protein 12 kDa (FKBP12) (Wong and Pessah 1997). Consistent with this mechanism, enhancement of [^3^H]Ry binding to RyR1 by BDE-4 (5 μM) was inhibited by inclusion of rapamycin in the assay (2 min preincubation) to disrupt the RyR1-FKBP12 complex (Figure 1D). The importance of the FKBP12 complex in mediating the efficacy of BDE-4 toward RyR1 was further verified by measuring active uptake and release of Ca^2+^ from the same membrane vesicles used for [^3^H]Ry binding analysis. Addition of BDE-4 (5 μM) to vesicles actively loaded with Ca^2+^ caused release of accumulated Ca^2+^, and this response was inhibited in vesicles pretreated with rapamycin in a concentration-dependent manner (Figure 1E,F). Rapamycin eliminated BDE-4–triggered Ca^2+^ release without inhibiting caffeine-induced Ca^2+^ release (Figure 1E), indicating that PBDEs sensitize RyR1 via a mechanism previously reported for PCBs.

### *Para* substitution of environmentally relevant PBDEs determines RyR1 and RyR2 activity

**Figure 2:**
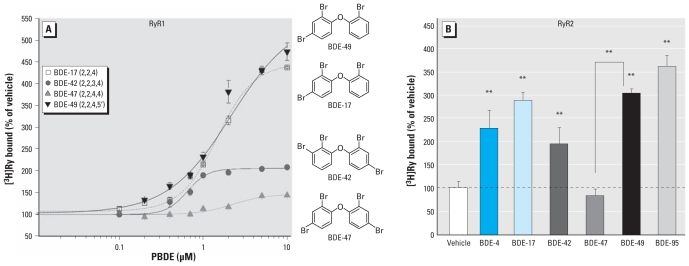
Substitution of both *para* positions with bromine reduces PBDE activity toward RyR1 and RyR2. (*A*) PBDEs possessing one *para*-bromine substituent (BDE-17, BDE-42, and BDE-49) enhanced [^3^H]Ry binding to RyR1 microsomes in a concentration-dependent manner (maximum of 446% for BDE-17, 206% for BDE-42, and 527% for BDE-49 compared with vehicle); in contrast, BDE-47, with two *para*-bromine substituents, showed significantly lower efficacy (maximum of 145% compared with vehicle). (*B*) SAR for PBDEs (each at 10 μM) as assessed using [^3^H]Ry binding analysis of microsomes enriched in RyR2; *n* = 3 or 4 independent experiments, each in triplicate. **p < 0.01.

Because *para*-chloro-substitutions of PCBs significantly influence activity toward RyR1 (Pessah et al. 2006), we investigated whether *para*-bromo-substitutions similarly influence the activity of PBDEs toward RyRs, using congeners with higher bromination that are environmentally relevant. We used a more activating basal buffer condition in these experiments to detect both activating and inhibiting actions of PBDEs on RyR activity. Figure 2A shows that BDE-47 weakly enhanced binding of [^3^H]Ry to RyR1 (maximum, ~ 145% of vehicle control at 10 μM), whereas BDE-49 and BDE-17 enhanced binding > 450% relative to vehicle control (EC_50_ = 2 μM). BDE-42 showed intermediate activity (250% of vehicle control at 10 μM) (Figure 2A). A similar SAR was observed with RyR2 (Figure 2B). At concentrations that maximally enhance RyR1, BDE-17 and BDE-49 (10 μM) increased [^3^H]Ry binding to RyR2 by approximately 300%, whereas BDE-42 was intermediate (200%), and BDE-47 had negligible activity. A similar SAR was identified with [^3^H]Ry binding to cortical membrane preparations that contain a mixture of RyR1 and RyR2 [see Supplemental Material, Figure 1 (doi:10.1289/ehp.1002728)]. Collectively, these data indicate that in addition to two *ortho*-bromines, one unsubstituted *para* position is critical for enhancing RyR activity.

### *Para*-methoxy diminishes but does not eliminate RyR1 activity

To further test the hypothesis that the *para* substitution is an important determinant of RyR activity, we examined the activity of *para-*methoxy metabolites using [^3^H]Ry binding and Ca^2+^ flux analyses of RyR1. Addition of a methoxy substituent at the 4 or 4′ positions of BDE-17, BDE-42, or BDE-49 significantly reduced the apparent efficacy by > 2-fold in [^3^H]Ry binding experiments [see Supplemental Material, Figure 2A (doi:10.1289/ehp.1002728)]. However, addition of methoxy to the 5 position of BDE-47 did not alter activity toward RyR1. Addition of BDE-49 (10 μM) subsequent to loading vesicles with Ca^2+^ in the presence of ATP elicited a very robust efflux of the accumulated Ca^2+^, and the release could be completely blocked by the RyR channel blocker RR (see Supplemental Material, Figure 2B, top). 4′-Methoxylated-BDE-49 (10 μM) added to the Ca^2+^-loaded vesicles under identical assay conditions elicited a much slower release of Ca^2+^ than the parent congener, and these effects were blocked by RR (see Supplemental Material, Figure 2B, middle). In contrast, BDE-47 (10 μM) did not sufficiently sensitize RyR1 to produce net Ca^2+^ efflux from the vesicles in the presence of the strong SERCA pump activity present in this assay (see Supplemental Material, Figure 2B, bottom).

### Rapamycin pretreatment selectively negates PCB-49–induced Ca^2+^ release

As predicted by results with BDE-4 (Figure 1B), microsomes preincubated with 20 μM rapamycin or vehicle 2 min before the Ca^2+^-loading phase greatly diminished subsequent response to BDE-49 (2 μM). However, regardless of the pretreatment protocol, the RyR1 channels remained responsive to 4-CmC [see Supplemental Material, Figure 3 (doi:10.1289/ehp.1002728)].

### Hydroxylation can differentially influence PBDE activity toward RyR1

**Figure 3:**
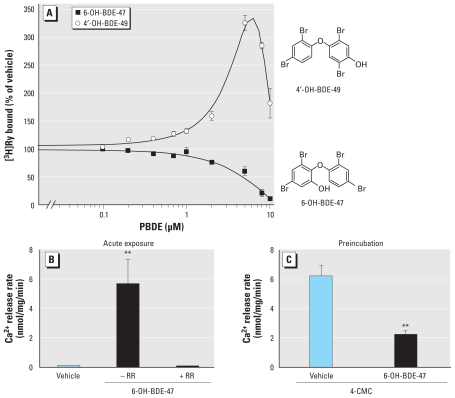
Complex activity of 6-OH-BDE-47 and 4′-OH-BDE-49 toward RyR1. (*A*) [^3^H]Ry binding analysis under equilibrium conditions (3 hr at 37°C) indicates that 6-OH-BDE-47 inhibits RyR1, whereas 4′-OH-BDE-49 alters [^3^H]Ry binding in a nonmonotonic manner (*n* = 3 independent experiments in triplicate). (*B*) Microsomal vesicles actively loaded with Ca^2+^ release their accumulated Ca^2+^ when acutely challenged with 6-OH-BDE-47 (10 μM), whereas RR (1 μM) blocked 6-OH-BDE-47–induced Ca^2+^ release (*n* = 3). (*C*) Preincubation (> 30 min) with 6-OH-BDE-47 significantly attenuates 4-CmC (1 mM)-induced Ca^2+^ release from microsomes (*n* = 3). **p < 0.01.

OH-BDE metabolites have been found in maternal and fetal blood (Qiu et al. 2009); therefore, we tested the activity of the environmentally relevant hydroxylated metabolites 6-OH-BDE-47 and 4′-OH-BDE-49 toward RyR1 using [^3^H]Ry binding analysis (Figure 3A). Unexpectedly, 4′-OH-BDE-49 exhibited a nonmonotonic concentration–effect relationship, activating RyR1 at approximately 350% of vehicle control at 5 μM but having reduced efficacy at higher concentrations (< 200% of vehicle control at 10 μM). Interestingly, 6-OH-BDE-47 inhibited [^3^H]Ry binding in a concentration-dependent manner, with complete inhibition observed at 10 μM (Figure 3A). Because the rate of [^3^H]Ry binding to RyRs is too slow to detect transient activation of RyR1 if it occurred, we proceeded to test whether 6-OH-BDE-47 had temporally distinct actions, initially activating and subsequently blocking RyR1 channel activity, by measuring the actions of this congener on microsomal Ca^2+^ fluxes. Indeed, microsomal vesicles rapidly released their accumulated Ca^2+^ when acutely challenged with 6-OH-BDE-47 (10 μM), and this effect was completely blocked by RR (Figure 3B). In addition, microsomal vesicles preincubated with 6-OH-BDE-47 at 37°C (< 2 hr) significantly inhibited 4-CmC–induced Ca^2+^ release (Figure 3C), indicating that prolonged incubation does subsequently block RyR1, as predicted from [^3^H]Ry binding studies (Figure 3A).

### Nanomolar BDE-49, but not BDE-47, sensitizes RyR-mediated Ca^2+^release in intact cells

To determine whether the different efficacies of BDE-47 and BDE-49 toward RyRs extend to RyR-dependent signaling events in intact cells, we tested the activity of the two congeners toward HEK293^null^ cells (which lack any expression of RyRs) and HEK293 cells, which stably express either RyR1 (HEK293^RyR1^) or RyR2 (HEK293^RyR2^) [see Supplemental Material, Figure 4A,D (doi:10.1289/ehp.1002728)]. Cells of each genotype were pretreated with 250 nM BDE-49 or BDE-47 for 16 hr before loading them with the Ca^2+^-sensitive dye Fluo-4. Once loaded with Ca^2+^ indicator, cells were imaged to detect changes in cytoplasmic Ca^2+^ ([Ca^2+^]_i_) before and after challenge with RyR agonists caffeine or 4-CmC (Fessenden et al. 2000). HEK293^RyR1^ responded to brief (10-sec) focal application of caffeine with a Ca^2+^ transient whose amplitude was concentration dependent (see Supplemental Material, Figure 4B). Pretreatment of HEK293^RyR1^ cells with BDE-49 enhanced caffeine-induced Ca^2+^ release, resulting in larger transient amplitudes than vehicle control (*p* < 0.05) at lower caffeine concentrations (see Supplemental Material, Figure 4B,C). In contrast, BDE-47 did not alter caffeine responses compared with vehicle control. HEK 293^null^ cells failed to respond to RyR1 agonists even when pretreated with BDE-49 (see Supplemental Material, Figure 4B). HEK293^RyR2^ cells responded vigorously to a brief puff of 4-CmC (1 mM) in contrast to HEK293^null^ cells (see Supplemental Material, Figure 4E). HEK293^RyR2^ cells pretreated for 16 hr with BDE-49, but not BDE-47, showed significantly larger Ca^2+^ transient amplitudes (~ 180%; *p* < 0.05) compared with vehicle controls (see Supplemental Material, Figure 4F).

### RyR activity predicts neurotoxic potential

**Figure 4:**
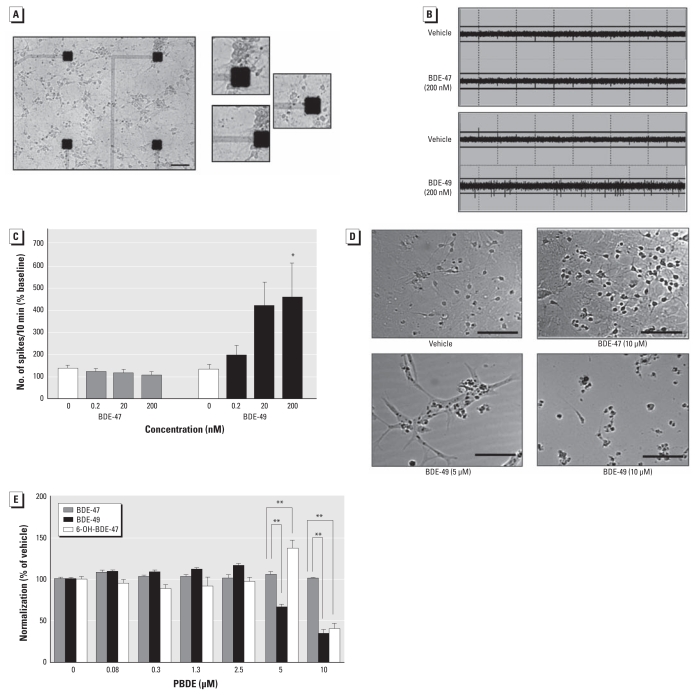
Cortical neurons show excitoxicity to BDE-49 and 6-OH-BDE-47, but not BDE-47. (*A*) Primary cultured rat cortical neurons on MEA (21 days *in vitro*); bar = 100 μm. (*B*) Representative raster plot of spike trains over a 6-sec period in neurons exposed acutely to vehicle, BDE-47, or BDE-49. (*C*) BDE-49, but not BDE-47, increases spontaneous spike activity in a concentration-dependent manner; data are presented as mean ± SE (*n* = three arrays per treatment group). (*D*) Micrographs of neurons after 48 hr exposure to vehicle, BDE-47 (10 μM), or BDE-49 (5 μM or 10 μM) between 6 and 8 days *in vitro*. Compared with neurons exposed to vehicle and BDE-47, BDE-49–exposed neurons showed pronounced morphological changes, including fasciculation and decreased soma diameter; bar = 150 μm. (*E*) BDE-49 (5 and 10 μM) and 6-OH-BDE-47 (10 μM), but not BDE-47, significantly decreased cell viability as assessed using the MTS assay. *p < 0.05 compared with vehicle control by ANOVA with post hoc Tukey’s test. **p < 0.01.

Considering the widely divergent activities of BDE-47 and BDE-49 on RyR1 and RyR2, both of which are expressed in brain, we tested whether their differential effects on RyR activity predict neurotoxicity. First, we examined how these PBDEs influence spontaneous electrical spiking activity of cortical neurons cultured on microelectrode arrays (MEA). MEAs have been used increasingly to assess altered development of neural networks and excitotoxicity caused by xenobiotics (Johnstone et al. 2010). Cortical neurons cultured on MEA probes exhibited spontaneous spike (action potential) activity at 21 days *in vitro* (Figure 4A,B). Sequential exposure to increasing concentrations of BDE-49 (0, 0.2, 20, and 200 nM) at 10-min intervals significantly increased the number of spontaneous spikes, whereas BDE-47 (< 200 nM) did not alter spike activity relative to the control period (Figure 4B,C).

We next determined whether exposures to BDE-47 and BDE-49 at higher concentrations (low micromolar) and for longer times (48 hr) influence the viability of cortical neuron cultures. Compared with vehicle controls, cultures exposed to 5 and 10 μM BDE-49 invariably showed loss of phase-bright cell bodies, decreased soma diameter, extreme fasciculation of processes, and a tendency for the monolayer to pull up off the substrate (Figure 4D). In contrast, no overt morphological abnormalities were observed in neurons exposed to BDE-47 (5 μM, 48 hr). Cell viability assessed using the MTS assay confirmed that BDE-49 (> 5 μM) significantly decreased neuronal cell viability by > 50% (*p* < 0.05). A potentially significant finding is that 6-OH-BDE-47 (10 μM), a major metabolite of BDE-47, also caused significant loss of cell viability (Figure 4E).

## Discussion

The relatively high levels of PBDEs recently measured in young children in North America (Herbstman et al. 2010), and especially California (Rose et al. 2010), escalates concerns about their effects on human and environmental health that were raised a decade ago (Darnerud et al. 2001). Levels of the most abundant congeners in serum—BDE-47, BDE-99, and BDE-100—in children < 72 months of age have been reported to be associated with lower scores on tests of cognitive, behavioral, and physical development (Herbstman et al. 2010). PBDE levels were also correlated with impairments in fine psychomotor abilities and attention but improved coordination, visual perception, and behavior (Roze et al. 2009). Similarly, *in utero* or postnatal exposure to PBDEs resulted in hyperactivity in rats (Kuriyama et al. 2005; Suvorov et al. 2009) and mice (Viberg et al. 2004). Exposure to PBDEs in neonatal mice resulted in learning deficits in visual discrimination tasks (Dufault et al. 2005).

Despite the mounting evidence that PBDEs cause developmental neurotoxicity, the principal molecular targets responsible for this toxicity have not been identified. PBDEs are usually found in combination with other persistent organic pollutants such as PCBs, triclosan, and *o*,*p*′-DDE (*o*,*p*′-dichlorodiphenyldichloroethylene), each of which has been shown to alter Ca^2+^ homeostasis, in part by dysregulating RyR channels (Ahn et al. 2008; Morisseau et al. 2009; Pessah et al. 2010). RyRs, along with inositol 1,4,5-trisphosphate receptors (IP_3_Rs), are a family of Ca^2+^ channels in endoplasmic or sarcoplasmic reticulum, broadly expressed in both excitable cells (striated muscles, neurons) and nonexcitable cells (dendritic cells, T lymphocytes) (Pessah et al. 2010). RyRs assemble as very large homotetrameric structures with a molecular weight > 2 MDa and consist of a very large cytoplasmic assembly (the foot region) and a relatively small transmembrane assembly composed of six putative transmembrane passes with a central ion-conducting pore. All three isoforms of RyRs are expressed in the central nervous system but are differentially distributed among specific brain regions, cell types, and cell regions, reflecting their participation in specialized functions (Pessah et al. 2010). Environmental toxicants that have a potential for altering RyR function can influence neuronal excitability, alter synaptic plasticity, and activate cytosolic and nuclear transcriptional events implicated in activity-dependent dendritic growth (Pessah et al. 2010).

The SAR of PBDEs toward RyR1 and RyR2 indicate that the location of *ortho*- and *para-*bromine substituents significantly influence the level of activation of RyRs, as highlighted by divergent activities of BDE-47 and BDE-49, and is unrelated to differences in lipophilicity. In this regard, the free concentration of BDE-49 is likely to be significantly lower than those added to the assays used in this study. This aspect of SAR is similar to that observed with the influence of *para*-chlorines on PCB activity toward RyR1 and RyR2 (Pessah et al. 2006). In this respect, the larger bromine substitution at the *para* positions more dramatically reduces PBDE activity than the corresponding chlorine substitutions on PCBs and may be because of steric interference. The similarity in structure–activities with *ortho* and *para* substitutions suggests a common mechanism for their effects on RyR activity. In support of a convergent mechanism, the activity of PCBs, bastadins, and PBDEs toward RyR1 can be selectively eliminated by using rapamycin to disrupt the RyR1-FKBP12 complex (Chen et al. 1999; Pessah et al. 2006; Wong and Pessah 1997), an accessory protein important in fine-tuning the gating properties of RyR1 channels (Brillantes et al. 1994).

The results from the present study showing that 6-OH-BDE-47 produces temporally defined biphasic actions on microsomal Ca^2+^ release is consistent with results obtained by Dingemans et al. (2008) showing that acute exposure of PC12 cells to 6-OH-BDE-47 promotes Ca^2+^ release from intracellular Ca^2+^ stores. [^3^H]Ry binding analysis reflects the activity of RyR channels at steady-state conditions for the radioligand (3 hr) and apparently fails to detect the initial channel activation elicited by 6-OH-BDE-47. However Ca^2+^ flux measurements clearly show that the immediate actions of this congener are activation of RyR1 and release of Ca^2+^ accumulated in microsomal vesicles, whereas prolonged exposure significantly attenuates 4-CmC–induced Ca^2+^ release. Our results reveal that OH-PBDEs have complex biphasic actions on RyR function and SR/ER Ca^2+^ transport properties that depend not only on the local concentration but also on the length of the exposure.

The complex interactions of OH-BDEs leading to activation or inhibition of RyR1 channels appear to mirror the complex SAR described with naturally occurring macrocyclic bromotyrosine toxins from *Ianthella basta,* termed “bastadins” (Chen et al. 1999; Mack et al. 1994). The reduced toxicophore that confers RyR activity resides within the eastern and western noncoplanar bromocatechol ether moieties that resemble OH-BDE (Masuno et al. 2006). The bromine and hydroxyl substituents about the diphenylether can result in either channel activation or channel inhibition.

Our results with OH-BDEs suggest an underlying mechanism for the results reported by Dingemans et al. (2008, 2010), who identified a number of 4-OH, 5-OH, and 6-OH metabolites of BDE-47 and BDE-49 that at 5–20 μM were more potent at mobilizing Ca^2+^ from ER and/or mitochondrial stores than their respective parent structures when applied to unstimulated (resting) PC12 cells. A high concentration of BDE-47 (20 μM) enhanced the Ca^2+^ transient amplitude triggered by bolus addition of extracellular K^+^, whereas other PBDE congeners lacked significant influence (Dingemans et al. 2010). Interestingly hydroxylation at the 4, 5, and 6 positions of BDE-47 or BDE-49 (2–20 μM) significantly inhibited depolarization-triggered Ca^2+^ transient amplitude. These previously reported effects of PBDEs and OH-PBDEs could be explained, at least in part, by their actions on RyR channels as we describe here. Sensitization of RyRs in resting cells by OH-PBDEs could result in chronic Ca^2+^ leakage and depletion of ER stores. This could account in large part for both the rapid and delayed rises in cytoplasmic Ca^2+^ previously observed by Dingemans et al. (2008, 2010). The suppression of the Ca^2+^ transient amplitude triggered by depolarization subsequent to 20 min pretreatment with 2 and 20 μM OH-PBDE (Dingemans et al. 2010) would be expected to cause partial or complete depletion of ER stores as a direct consequence of submaximal or maximal activation of RyR channels, respectively. Interestingly, 4-OH-BDE-49 shows a nonmonotonic concentration–response relationship toward RyR1. In contrast, BDE-49 is a very efficacious activator of RyRs in [^3^H]Ry binding studies, potently sensitizes caffeine- or 4-CmC–triggered Ca^2+^ release in HEK293 cells that express RyRs, and is a potent excitotoxicant toward primary cortical neurons. In this regard, 48 hr exposure to BDE-47 < 10 μM does not promote loss of neuronal viability, whereas BDE-49 does. These observations are apparently at odds with reports that developmental exposure to BDE-47 causes developmental neurotoxicity in rodent models (Dingemans et al. 2007; Suvorov et al. 2009). One interpretation of this discrepancy is that RyR-independent mechanisms mediate the adverse effects of BDE-47 on the developing nervous system. An alternative suggestion supported by our data is that the neurotoxic effects of BDE-47 observed *in vivo* are mediated by 6-OH-BDE-47 rather than the parent congener; the lack of effect of the parent compound on either spontaneous activity or cell viability in cultured cortical neurons reflects the limited metabolic capacity of this *in vitro* model.

The present study indicates that [^3^H]Ry binding studies are a rapid means of defining SARs that predict neurotoxic PBDEs, much like its use to identify neurotoxic PCBs (Pessah et al. 2010). The toxicological significance of these findings is illustrated by a recent report by (Miller et al. 2009) that BDE-49, which is not typically measured in human samples, was detected in gestational tissues from women in southeast Michigan at levels comparable with BDE-47. BDE-49 comprised 17% of the total PBDE concentration in these tissues. This observation is consistent with reports identifying BDE-49 as a major contributor to PBDE load in fish (Mariottini et al. 2008; Roosens et al. 2008), including one study of Great Lakes fish that identified BDE-49 as the most abundant congener (Manchester-Neesvig et al. 2001). The mechanisms contributing to this apparent selective enrichment of BDE-49 have yet to be determined, but as discussed by Miller et al. (2009), these findings suggest that the majority of human studies underestimate PBDE levels by as much as 14–19%. Our studies suggest that the problem is even greater, in that most exposure studies fail to account for congeners that pose significant risk to the developing nervous system. The SAR defined in our study provides a tool for refining human exposure studies to focus on those PBDE congeners with the greatest neurotoxic potential.

## Conclusions

The present study is the first to identify RyR1 and RyR2 as direct targets of both PBDEs and their hydroxylated metabolites. These results are significant because RyRs are broadly expressed in excitable and nonexcitable cells, where they regulate key physiological and pathophysiological functions (Pessah et al. 2010). Certain PBDEs and their hydroxylated metabolites have potent (maximum activities < 10 μM) and high efficacies toward altering the activities of RyR1 and RyR2 channels, and these same PBDEs are found in maternal and fetal blood, and human gestational tissues (Miller et al. 2009; Qiu et al. 2009) as well as in animal tissues (Marsh et al. 2004; Qiu et al. 2007; Valters et al. 2005). In addition, the known contribution of RyRs to inherited and acquired disorders (Pessah et al. 2010) strongly suggests that Ca^2+^ signaling dysregulation mediated by RyR channel mechanisms should be included in assessments of risk.
