# Erratum: “*Para*– and *Ortho*-Substitutions Are Key Determinants of Polybrominated Diphenyl Ether Activity toward Ryanodine Receptors and Neurotoxicity”

**DOI:** 10.1289/ehp.120-A189

**Published:** 2012-05-01

**Authors:** Kyung Ho Kim, Diptiman D. Bose, Atefeh Ghogha, Joyce Riehl, Rui Zhang, Christopher D. Barnhart, Pamela J. Lein, Isaac N. Pessah

**Affiliations:** 1 Department of Molecular Biosciences, School of Veterinary Medicine, and; 2 Center for Children’s Environmental Health, University of California–Davis, Davis, California, USA

NOTE: In Figure 4B of the article “*Para*- and *Ortho*-Substitutions Are Key Determinants of Polybrominated Diphenyl Ether Activity toward Ryanodine Receptors and Neurotoxicity” by Kim et al. [Environ Health Perspect 119:519–526 (2011)], the authors inadvertently included the same representative trace for the BDE-47 exposure (second trace in Figure 4B) as for the vehicle exposure (first trace).

The authors apologize for the error.

This error has been corrected in the PDF version of this article.

